# Supplementary material for: Chip-integrated van der Waals PN heterojunction photodetector with low dark current and high responsivity
Source: Light Sci Appl. 2022 Apr 20;11:101. doi: 10.1038/s41377-022-00784-x (PMC9021258; doi:10.1038/s41377-022-00784-x)
Supplement: Supplementary file 1 — Supplemental Material for Chip-integrated van der Waals PN heterojunction photodetector with low dark current and high responsivity [file 41377_2022_784_MOESM1_ESM.docx]

**Supplementary Information for**

**Chip-integrated van der Waals PN heterojunction photodetector with low dark current and high responsivity**

Ruijuan Tian^1^, Xuetao Gan^1,^*, Chen Li^1^, Xiaoqing Chen^1^, Siqi Hu^1^, Linpeng Gu^1^, Dries Van Thourhout^2^, Andres Castellanos-Gomez^3^, Zhipei Sun^4^, Jianlin Zhao^1^

1. Key Laboratory of Light Field Manipulation and Information Acquisition, Ministry of Industry and Information Technology, and Shaanxi Key Laboratory of Optical Information Technology, School of Physical Science and Technology, Northwestern Polytechnical University, 710129 Xi’an, China
2. Photonics Research Group, Center for Nano and Biophotonics, Ghent University, B-9000 Gent, Belgium
3. Materials Science Factory, Instituto de Ciencia de Materiales de Madrid (ICMM-CSIC), E-28049 Madrid, Spain
4. Department of Micro- and Nanosciences, Aalto University, Tietotie 3, FI-00076 Espoo, Finland

**Email:* xuetaogan@nwpu.edu.cn

**Fig. S1** Raman spectra of the BP, MoTe_2_, and BP/MoTe_2_ heterostructure.

Raman spectra from the BP, MoTe_2_ and their heterojunction region are presented in Fig. S1. The observed Raman-active modes of BP and MoTe_2_ are consistent with the previous reports^S1^. The peaks of both BP and MoTe_2_ can be observed in the overlapped region, indicating high qualities of materials in the heterojunction region after mechanical exfoliation and device fabrication.

**Fig. S2** **a-d** Atomic force microscopy (AFM) measurement results of the the bottom h-BN (a), BP (b), MoTe_2_ (c) and the top h-BN (d) layers employed to construct the chip-integrated van der Waals heterostructure, which has thicknesses of 29.6, 13, 10.6 and 28.5 nm, respectively. Inset: AFM images and scanning length marked by the white dotted line.

**Fig. S3** **a** Diagram of the Mach-Zenhder interferometer (MZI) with an absorption material on the long arm. **b** Normalized transmission spectra of the fabricated MZI measured after each step of the 2D material transfer during the fabrication, which have been vertically translated for clarification. From the extinction ratios (ERs), the absorption coefficient of each transferred layer can be determined. Each absorption coefficient represents the absorption characteristics of new added material when the individual material is transferred. Using this method, the absorption coefficient of the BP in the completed device is estimated as 0.09815 dB μm^−1^. **c** Transmission spectrum of fabricated MZI before the integration of 2D materials, where the incident laser power is fixed during its wavelength scanning from 1500 to 1630 nm. It shows the coupling efficiency of the grating couplers is reduced gradually for long wavelength range.

To precisely measure the optical absorption by the integrated 2D materials, we employed an unbalanced Mach-Zenhder interferometer (MZI) and placed the BP/MoTe_2_ heterostructure on one of the two arms. The schematic diagram of the proposed device is shown in Fig. S3a, which has an absorption material on the long arm. The presence of absorption in one of the arms will cause partial interference at the output spectrum of the MZI. By measuring the extinction ratio (*ER*) of the interference fringes, the absorption in the lossy arm can be extracted. We assume the coupling coefficient of the couple-in and couple-out grating couplers at the two ends of the MZI are the same, which is defined as *η*_grating_ here. By measuring the incident laser power *P*_1_, the output power *P*_2_, and *ER* of the interference fringes from the MZI, the coupling coefficient *η*_grating_ could be calculated by^S2^

$\eta_{\text{grating}}=\sqrt{\frac{\max(P_{2})(1+2\sqrt{1+2\sqrt{ER}+ER})}{P_{1}ER}}$ S(1)

Here, *ER* in terms of the absorption in long arm is expressed as^S3^

$ER=\frac{P_{\max}}{P_{\min}}=\frac{1+2e^{-\alpha L/2}+e^{-\alpha L}}{1-2e^{-\alpha L/2}+e^{-\alpha L}}=\left(\frac{1+e^{-\alpha L/2}}{1-e^{-\alpha L/2}}\right)^{2}$ S(2)

where *α* is the optical power absorbed by the absorption materials integrated on the waveguide per unit length (in μm^−1^), normalized to the power transmitted in the waveguide. If we multiply *α* by 10 log10(e), we can convert it to dB μm^−1^. From above, we can precisely extract the absorption coefficient of the absorption material integrated on the MZI. The absorption coefficient *α*_m_ of each material layer integrated on the MZI can be specifically obtained as follows

$\alpha_{\text{m}}=\frac{1}{l_{\text{m}}}\left[In\left(\frac{\sqrt{P_{\text{max}}/P_{\min}}+1}{\sqrt{P_{\max}/P_{\min}}-1}\right)-\alpha_{0}l_{0}-\alpha_{1}l_{1}-\alpha_{2}l_{2}-\alpha_{\text{m-1}}l_{\text{m-1}}\right]$ S(3)

Finally, the absorption optical power *P*_m_ of the each added materials on the MZI could be acquired by

$P_{\text{m}}=\frac{P_{\text{in}}\cdot\eta_{\text{GC}_{\text{m}}}}{2}\left(1-e^{-2\cdot\alpha_{\text{m}}\cdot\Delta l_{\text{m}}}\right)$ S(4)

Figure S3b presents the normalized transmission spectra of the MZI during each step of material transfer. The *ER* can be obtained from the interference fringes in the spectra. The absorption loss in the measurement arm of the MZI can be determined using Equation S(1), S(2), S(3) and S(4). Note the absorption coefficients can be overestimated due to the influences of reflection and scattering losses occurring in the 2D material-coated waveguide region, which could result in an underestimated responsivity of the device. This provides a direct relation between a measurable quantity *ER* and the absorption coefficient, which is independent on the input power or the grating coupler efficiency. This is a great advantage over other methods where the coupling efficiency is often estimated from measurements of a reference waveguide, which unfortunately varies significantly from different devices considering the fabrication errors and coupling configurations.

In Fig. S3c, we also plot the transmission spectrum the fabricated MZI before the integration of 2D materials. By fixing the laser power and scanning the laser wavelength from 1500 to 1630 nm, the transmission powers reduce gradually, which could be attributed to the degraded coupling efficiency at the longer wavelength range for the two grating couplers at the two ends of the MZI^S4^. This could be used to explain the wavelength dependence of the photocurrents shown in Fig. 3a of the main text, which gradually decreases for longer wavelength with a constant incident laser power.

To evaluate the optical absorption of BP on the waveguide, we performed the guiding mode analysis of the silicon nitride waveguide integrated with a BP flake using the FEM software COMSOL. The silicon nitride waveguide with a thickness of 300 nm and width of 1080 nm supports a quasi-TE mode as expected. A BP flake with a thickness of 13 nm is integrated on the waveguide, which has a complex refractive index of *n*_xx_ = 3.16+0.0623i, *n*_yy_ = 2.83, *n*_zz_ = 3.64+0.135i^S3^. Figure S4 shows the mode profile of the BP-integrated waveguide. From the corresponding complex effective index of the mode, the absorption coefficient of the BP flake is estimated as 0.0911 dB μm^−1^, which is consistent with the experimentally extracted absorption (0.0982 dB μm^–1^) for the 13 nm thick BP.


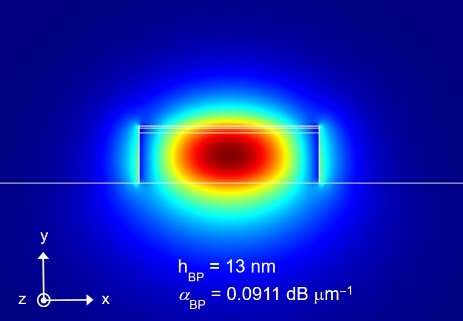


**Fig. S4** Electric field distribution of the TE fundamental mode for the silicon nitride waveguide coated with a 13 nm thick BP flake, presenting a calculated BP absorption coefficient of 0.0911 dB μm^–1^.

**Supplementary Table 1** Comparisons of figure-of-merit for chip-integrated 2D material photodetectors

| **2D Materials** | **Responsivity**  **(mA W^−1^)** | ***I*_dark_**  **(A)** | ***f*_3 dB_**  **(Hz)** | **Spectral Range**  **(nm)** | **Ref.** |
| --- | --- | --- | --- | --- | --- |
| Graphene  (M-S-M) | 15.7 (*V*_DS_ = 0 V) | – | 2×10^10^ | 1550 | ^S5^ |
| Graphene  (G-S-G) | 50 (*V*_DS_ = 0 V) | – | 1.8×10^10^ | 1550 | ^S6^ |
| Graphene  (PN junction) | 76 (*V*_DS_ = 0.3 V)  35 (*V*_DS_ = 0 V) | 5×10^5^  @0.3V  (*V*_g_) | 6.5×10^10^ | 1550 | ^S7^ |
| Graphene  (G-S-G) | 15 (*V*_DS_ = 0 V) | – | 3×10^10^ | 1550 | ^S8^ |
| Graphene  (M-G-Si) | 85 (*V*_DS_ = −1 V)  370 (*V*_DS_ = 3 V) | 2×10^−8^  >1×10^−6^ | – | 1550 | ^S9^ |
| Graphene/MoS_2_ | 240 (*V*_DS_ = 10 V) | 1.3×10^−8^ | 2.8×10^10^ | 1550 | ^S10^ |
| Graphene  (plasmonic enhanced) | 70 (*V*_DS_ = −0.3 V)  400 (*V*_DS_ = −0.3 V) | – | >2×10^10^  >4×10^10^ | 2000  1500 | ^S11^ |
| BP(PV, PB) | 135 (11.5 nm)  (*V*_DS_ = −0.4 V)  657 (100 nm) (*V*_DS_ = 2 V) | 2.2×10^−7^  5.6×10^−4^ | 3×10^9^ | 1550 | ^S3^ |
| BP(PC, PG) | 10^3^ (60 nm) | >9×10^−6^ | 1.5×10^5^ | 1550 | ^S12^ |
| BP | 306.7 (*V*_DS_ = 0.4 V) | – | 1.33×10^9^ | 2000 | ^S13^ |
| BP | 40 (*V*_DS_ = 0.2 V) | 3.5×10^−5^ | – | 2185 | ^S14^ |
| BP | 2.3×10^4^ (*V*_DS_ = 1 V) | >1×10^−6^ | – | 3680-4030 | ^S15^ |
| BP | 1.13×10^4^ (*V*_DS_ = 0.5 V) | 3.7×10^−7^ | 1.64×10^3^ | 3800 | ^S16^ |
| MoTe_2_  (M-S-M) | 400 (*V*_DS_ = −3 V) | 2×10^−8^ | 5×10^8^ | 1310 | ^S17^ |
| MoTe_2_  (M-S-M) | 500 (*V*_DS_ = −2 V) (60 nm)  10 (*V*_DS_ = −2 V) (40 nm) | 1.3×10^−8^ | 3.5×10^7^ | 1550 | ^S18^ |
| MoTe_2_/Graphene  (PV, PC) | 200 (*V*_DS_ = −3 V) | 10^−6^ | 5×10^10^ | 1300 | ^S19^ |
| **BP/MoTe_2_** | **~277 (*V*_DS_ = 0)**  **397 (*V*_DS_ = 0 V, *V*_G_ = 60 V)**  **709 (*V*_DS_ = −1 V, *V*_G_ = 60 V)** | **6.8×10^−9^**  **2.32×10^−11^**  **1.7×10^−12^** | **1×10^9^** | **1550** | **This work** |

The following table serves as a comparison of the waveguide-integrated van der Waals PN heterojunction photodetector with the SiGe waveguide photodetectors.

**Supplementary Table 2** Comparison of our work with the reported SiGe waveguide photodetectors.

| **Materials** | **Wavelength Range** | **Responsivity** | **I_dark_** | **Response speed** | **Ref.** |
| --- | --- | --- | --- | --- | --- |
| Ge-SiGe | 1440-1530 nm | 0.17 A W^−1^ | 17.9 mA cm^−2^ | 2.5 Gb s^−1^ | ^S20^ |
| Ge | 1500-1620 nm | 0.89 A W^−1^ @−2 V | 1.69×10^−7^ A | 31.3 GHz | ^S21^ |
| GeSi | 1550 nm | >1 A W^−1^ @−3.3 V | − | >35 GHz | ^S22^ |
| Ge | 1520-1610 nm | 1 A W^−1^ @−4 V | 60 mA cm^−2^ | 42 GHz | ^S23^ |
| BP/MoTe_2_ | 1500-1630 nm | ~0.277 A W^−1^ (*V*_DS_ = 0)  0.397 A W^−1^  (*V*_DS_ = 0 V, *V*_G_ = 60 V)  0.709 A W^−1^  (*V*_DS_ = −1 V, *V*_G_ = 60 V) | 6.8×10^−9^ A  2.32×10^−11^ A  1.7×10^−12^ A | 1.0 GHz | This work |

We compare the performance of our waveguide-integrated BP/MoTe_2_ photodetector with the reported SiGe waveguide photodetectors, as shown in Supplementary Table 2. All of these photodetectors were proposed to provide chip-integrated photodetectors for silicon photonics. The comparisons in the characteristics of these photodetectors are described as follows: For the photoresponse spectral range, because the employed BP has a narrow bandgap around 0.3 eV, the proposed chip-integrated BP/MoTe_2_ van der Waals PN heterojunction photodetector could operate in the whole telecommunication band, which performs same as the SiGe photodetectors. For the responsivity of 0.277 A W^−1^ to 0.709 A W^−1^ obtained in our chip-integrated BP/MoTe_2_ PN heterojunction photodetector, it is on par with those of SiGe waveguide photodetectors. The dark current in our proposed photodetector is in the range of 6.8 nA to 1.7 pA, which is more than two to five orders of magnitude lower than those reported SiGe waveguide photodetectors. Although the measured response speed in our device is currently slower than the reported SiGe waveguide photodetetors, it has a large space to be improved, such as the optimization of the device structure^S19^. In addition, compared to the complicated process of the SiGe photodetector, the device fabrication process of our waveguide-integrated BP/MoTe_2_ photodetector is much simpler and cheaper.

**Fig. S5** *I*_DS_−*V*_DS_ curves measured from the van der Waals BP/MoTe_2_ PN heterojunction in dark and at varied absorption powers, which is a linear scale form of the data shown in Fig. 2a of the main text. An open-circuit voltage *V*_oc_ of 228 mV is obtained with a BP absorption power of 12.79 μW (orange line). Here, an optical power of 60.5 μW is coupled into the waveguide by the grating coupler with a coupling efficiency of 6.9 dB.


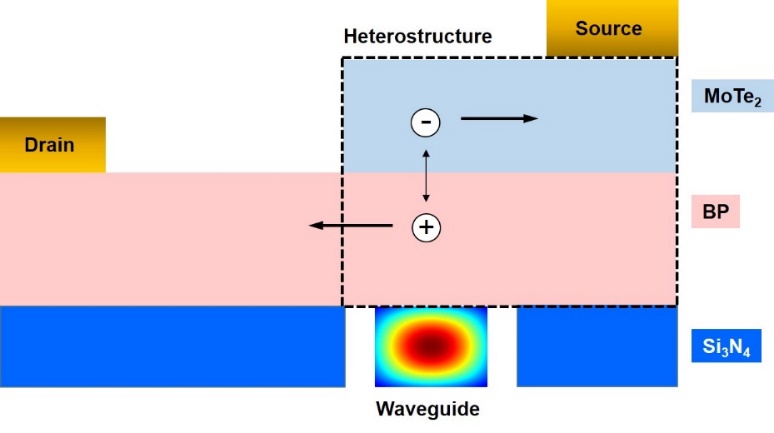


**Fig. S6** Schematic of photocurrent generation in the BP/MoTe_2_ heterostructure photodetector on waveguide under illumination. When an external electric field is applied to the BP/MoTe_2_ photodetector, the photogenerated electrons in the heterojunction region can be collected by one electrode. And photogenerated holes in the heterojunction must transport a long distance of the BP channel to reach the drain electrode.

**Fig. S7** **a** Fourier transformations of the measured impulse responses shown in Fig. 3b of the main text. **b** Experimental setup for measuring the dynamic response of the waveguide-integrated BP/MoTe_2_ heterojunction photodetector.

In our experiment, the measurement setup for the response speed of the presented BP/MoTe_2_ heterostructure waveguide photodetector is shown in Fig. S7b. A pulsed laser with ~5 ps pulse width, 100 MHz repetition rate, and wavelength of 1550 nm is chosen to provide the optical pulse chain. By coupling it into the waveguide-integrated van der Waals PN heterojunction photodetector, the resulting photoresponse in the form of electrical pulses was then extracted by using a Bias-Tee (Anritsu, G4N37, 8 kHz-40 GHz), which was also employed to supply a DC electrical bias over the detector. An electrical amplifier (Mini-circuits, ZKL-1R5+, 10-1500 MHz) was used to amplify the extracted pulsed electrical pulses. Finally, the amplified electrical signal was then transmitted into an oscilloscope (Lecroy, 740Zi-A) to estimate the frequency response.

**Fig. S8** Extracted rectification ratio at *V*_DS_ *=* +1/−1 V from Fig. 4a.


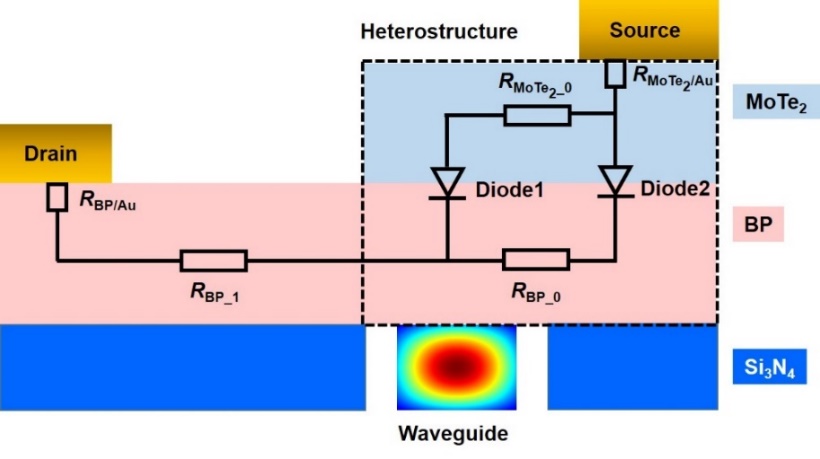


**Fig. S9** Schematics of the simplified equivalent circuit model of the waveguide-integrated BP/MoTe_2_ heterojunction.

**Fig. S10** **a** Optical microscope image of another fabricated devices, where D1 and D2 (S1 and S2) indicate the metal contacts for the BP (MoTe_2_) channel. Consequently, MoTe_2_ and BP field effect transistors (FETs) are formed on the silicon nitride slab with the back silicon substrate as the global gate electrode. Scale bar: 50 µm. **b** Transfer curve of the MoTe_2_ FET measured between S1 and S2 at *V*_DS_ = 1 V. **c** Transfer curve of the BP FET measured between D1 and D2 at *V*_DS_ = 1 V. The solid curves are in linear scale and the dashed curves are in semi-log scale.

**Fig. S11** Extracted series resistance as a function of the gate voltage by fitting the *I*_DS_-*V*_DS_ curves of Fig. 4a using the Shockley diode equation.

**Fig. S12** **a, b** Photocurrents as a function of the absorption power at (a) *V*_DS_ = 0 V and (b) *V*_DS_ = −1 V under different back gate voltages. **c** Responsivities of the device as a function of the back gate voltages at *V*_DS_ = 0 V and *V*_DS_ = −1 V. (d) Wavelength-dependent photocurrents of the device, where the incident light has a constant power and wavelength scanning range between 1500 and1630 nm.

**References**

S1. Xie, Y. *et al.* Gate-tunable photodetection/voltaic device based on BP/MoTe_2_ heterostructure. *ACS Applied Materials & Interfaces* **11**, 14215–14221 (2019).

S2. Youngblood, N. Waveguide integrated optoelectronics using two-dimensional materials. *Minneapolis：University of Minnesota* 38–43 (2016).

S3. Youngblood, N. *et al.* Waveguide-integrated black phosphorus photodetector with high responsivity and low dark current. *Nature Photonics* **9**, 247–252 (2015).

S4. Mekis, A. *et al.* A grating-coupler-enabled CMOS photonics platform. *IEEE Journal of Selected Topics in Quantum Electronics* **17**, 597–608 (2011).

S5. Gan, X. T. *et al.* Chip-integrated ultrafast graphene photodetector with high responsivity. *Nature Photonics* **7**, 883–887 (2013).

S6. Pospischil, A. *et al.* CMOS-compatible graphene photodetector covering all optical communication bands. *Nature Photonics* **7**, 892–896 (2013).

S7. Schuler, S. *et al.* Controlled generation of a p-n junction in a waveguide integrated graphene photodetector. *Nano Letters* **16**, 7107–7112 (2016).

S8. Gao, Y. *et al.* High-performance chemical vapor deposited graphene-on-silicon nitride waveguide photodetectors. *Optics Letters* **43**, 1399–1402 (2018).

S9. Goykhman, I. *et al.* On-Chip Integrated, Silicon-Graphene Plasmonic Schottky Photodetector with High Responsivity and Avalanche Photogain. *Nano Letters* **16**, 3005–3013 (2016).

S10. Gao, Y. *et al*. High-speed van der Waals heterostructure tunneling photodiodes integrated on silicon nitride waveguides. *Optica* **6**, 514–517 (2019).

S11. Guo, J. S. *et al.* High-performance silicon-graphene hybrid plasmonic waveguide photodetectors beyond 1.55 μm. *Light: Science & Applications* **9**, 29 (2020).

S12. Chen, C. *et al.* Three-Dimensional Integration of Black Phosphorus Photodetector with Silicon Photonics and Nanoplasmonics. *Nano Letters* **17**, 985–991 (2017).

S13. Yin, Y. L. *et al.* High-speed and high-responsivity hybrid silicon/black-phosphorus waveguide photodetectors at 2 µm. *Laser & Photonics Reviews* **13**, 1900032 (2019).

S14. Deckoff-Jones, S. *et al.* Chalcogenide glass waveguide-integrated black phosphorus mid-infrared photodetectors. *Journal of Optics* **20**, 44004 (2018).

S15. Huang, L. *et al.* Waveguide-integrated black phosphorus photodetector for mid-infrared applications. *ACS Nano* **13**, 913–921 (2019).

S16. Ma, Y. M. *et al.* High-responsivity mid-infrared black phosphorus slow light waveguide photodetector. *Advanced Optical Materials* **8**, 2000337 (2020).

S17. Ma, P. *et al.* Fast MoTe_2_ waveguide photodetector with high sensitivity at telecommunication wavelengths. *ACS Photonics* **5**, 1846–1852 (2018).

S18. Maiti, R. *et al.* Strain-engineered high-responsivity MoTe_2_ photodetector for silicon photonic integrated circuits. *Nature Photonics* **14**, 578–584 (2020).

S19. Flöry, N. *et al.* Waveguide-integrated van der Waals heterostructure photodetector at telecom wavelengths with high speed and high responsivity. *Nature Nanotechnology* **15**, 118–124 (2020).

S20. Fidaner, O. *et al.* Ge-SiGe quantum-well waveguide photodetectors on silicon for the near-infrared. *IEEE Photonics Technology Letters* **19**, 1631–1633 (2007).

S21. Yin, T. *et al.* 31 GHz Ge *n*-*i*-*p* waveguide photodetectors on Silicon-on-Insulator substrate. *Optics Express* **15**, 13965 (2007).

S22. Liu, J. F. *et al.* Design of monolithically integrated GeSi electro-absorption modulators and photodetectors on a SOI platform. *Optics Express* **15**, 623–628 (2007).

S23. Vivien, L. *et al.* 42 GHz p.i.n Germanium photodetector integrated in a silicon-on-insulator waveguide. *Optics Express* **17**, 6252–6257 (2009).
